# Anti-cancer effect of novel PAK1 inhibitor via induction of PUMA-mediated cell death and p21-mediated cell cycle arrest

**DOI:** 10.18632/oncotarget.15783

**Published:** 2017-02-28

**Authors:** Tae-Gyun Woo, Min-Ho Yoon, Shin-Deok Hong, Jiyun Choi, Nam-Chul Ha, Hokeun Sun, Bum-Joon Park

**Affiliations:** ^1^ Department of Molecular Biology, Pusan National University, Busan, Republic of Korea; ^2^ Department of Statistics, Pusan National University, Busan, Republic of Korea; ^3^ College of Agriculture and Life Sciences, Seoul National University, Seoul, Republic of Korea

**Keywords:** PAK1, anti-cancer, p21, PUMA, Bcl-2

## Abstract

Hyper-activation of PAK1 (p21-activated kinase 1) is frequently observed in human cancer and speculated as a target of novel anti-tumor drug. In previous, we also showed that PAK1 is highly activated in the Smad4-deficient condition and suppresses PUMA (p53 upregulated modulator of apoptosis) through direct binding and phosphorylation. On the basis of this result, we have tried to find novel PAK1-PUMA binding inhibitors. Through ELISA-based blind chemical library screening, we isolated single compound, IPP-14 (IPP; Inhibitor of PAK1-PUMA), which selectively blocks the PAK1-PUMA binding and also suppresses cell proliferation via PUMA-dependent manner. Indeed, in PUMA-deficient cells, this chemical did not show anti-proliferating effect. This chemical possessed very strong PAK1 inhibition activity that it suppressed BAD (Bcl-2-asoociated death promoter) phosphorylation and meta-phase arrest via Aurora kinase inactivation in lower concentration than that of previous PAK1 kinase, FRAX486 and AG879. Moreover, our chemical obviously induced p21/WAF1/CIP1 (Cyclin-dependent kinase inhibitor 1A) expression by releasing from Bcl-2 (B-cell lymphoma-2) and by inhibition of AKT-mediated p21 suppression. Considering our result, IPP-14 and its derivatives would be possible candidates for PAK1 and p21 induction targeted anti-cancer drug.

## INTRODUCTION

Elevated expression of PAK1 (p21-activated kinase 1) is frequently observed in various kinds of human cancers including colon or pancreatic cancers [1, 2]. This kinase has been reported to be involved in broad oncogenic properties including anti-apoptosis, cell cycle promotion and metastasis. Indeed, PAK1, activated by small GTP-proteins (such as cdc42 or Rac), can promote cell migration [3, 4], and can suppress the apoptosis through BAD phosphorylation [5–7]. In addition, PAK1-mediated Aurora A phosphorylation has been suggested as mitosis promoting mechanism [8–10].

In previous results, we revealed that activation of PAK1 in Smad4 deficient cancers suppresses PUMA-mediated apoptosis [11]. Actually, Smad4-deficient cancer cells are resistant to serum-deprivation-induced cell death [11]. Considering that half of pancreatic cancers and more than 20% of colon cancers show deletion of Smad4 [12–14], inhibition of PAK1 would be one of plausible strategy for treatment of human cancers such as pancreatic cancer and colon cancer.

In NF2 syndrome (neurofibromatosis type 2 syndrome), elevated PAK1 activity has been reported, because NF2 is inhibitor of PAK1 [15, 16]. According to recent report, NF2 suppresses PAK1 activity through direct interaction of regulatory domain [15]. Thus, selective PAK1 inhibitor has been proposed to be potent candidate target for NF2 syndrome as well as NF2 deficient human cancers such as mesothelioma [17]. In addition, PAK1 and AKT can activate each other through direct interaction [18]. Since AKT signaling is essential for cancer growth, PAK1 inhibition would also suppress the AKT activity.

Considering various role of PAK1 in cancer progression and initiation, PAK1 is very attractive target protein for development of anti-cancer drug. In this study, we have tried to find novel PAK1 inhibitory chemicals. On the basis of PAK1-PUMA binding, we have revealed that single novel compound can suppress PAK1 activity and induce cell death only in PAK1 activated and Bcl-2 overexpressed cancer cells. In addition, this chemical induces p21 and G2/M arrest. Thus, this chemical would be one of strong candidate for anti-cancer drug against various human malignancies including pancreatic cancers, colon cancer as well as NF2 deficient cancers.

## RESULTS

### Identification of PAK1-PUMA binding inhibitor

**Figure 1:**
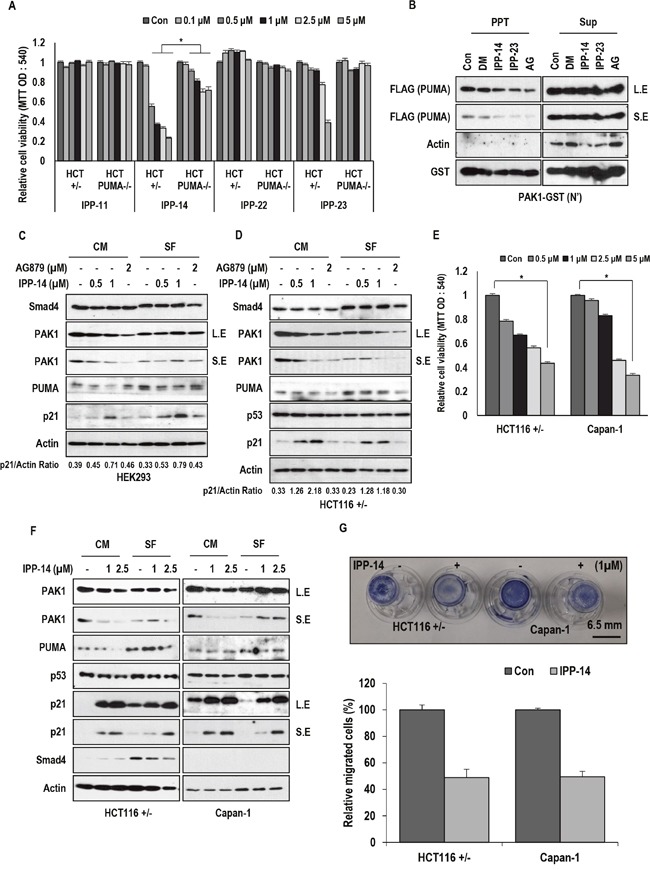
Isolation of PAK1 inhibitor **(A)** IPP-14 induces cell death in HCT116 cell lines but not in HCT PUMA deficient cells. IPP-14 reduces cell viability in HCT116 cells more effectively than other IPP. Following treatment with the indicated concentration of IPP for 48 hr, cell viability was measured by MTT assay. **(B)** PAK1-PUMA binding is inhibited by indicated chemicals. (DM; DMSO, IPP-14, 23 (5 μM), AG: AG879 (0.1 mM); known as PAK1 inhibitor). GST-PAK1 pull-down assay was performed with HEK293 (human embryonic kidney cells) cell lysates overexpressing PUMA-FLAG. Actin was used as loading control and negative control. PPT indicates precipitated proteins and Sup indicates supernatant. L.E indicates long exposure and S.E indicates short exposure. **(C)** IPP-14 induces p21 without obvious alteration of PAK1, Smad4 and PUMA in HEK293 cells. Cells were incubated with indicated chemicals for 8 hr with or without serum. p21/Actin ratio was measured by using Image J software. **(D)** IPP-14 shows similar effect in HCT116 cells. HCT116 cells were treated with same condition with HEK293 cells. In addition, p53 expression was not altered by IPP-14 despite p21 induction. Western blot was performed by using the indicated antibodies. Actin was used for loading control. p21/Actin ratio was measured by using Image J software. **(E)** IPP-14 suppresses cell viability of Capan-1, Smad4-deficient pancreatic cell line. The MTT assay was performed to measure cell viability following treatment of IPP-14 for 48 hr. **(F)** IPP-14 induces p21 in Capan-1, like as HCT116. HCT116 and Capan-1 cells were treated with indicating doses of IPP-14 in serum containing or deprivation conditions for 8 hr. Actin was used for loading control. **(G)** IPP-14 suppresses cell migration through PAK1 inhibition. HCT116, Capan-1 cells migration was monitored by using transwell assay with or without IPP-14 (1 μM) for 16 hr. The migration rate was quantified by counting the migration cells in six random fields. *P< 0.005 (t-test). The statistical significance between two groups was analyzed by Student's *t*-test. For all data sets, *P*< 0.05 was considered to be statistical significant.

In previous result, we revealed that serum-starvation-induced cell death can be activated by PUMA via Smad4-induced PAK1 inhibition. In fact, PAK1 blocks PUMA through direct binding and phosphorylation [11]. Thus, specific binding inhibitor of PAK1-PUMA would induce cell death in Smad4-deficient or PAK1 activated cancers. To prove this, we designed the ELISA (Enzyme-linked immunosorbent assay) based drug screening system using recombinant proteins [19] and performed the blind screening using 3 kinds of chemical libraries (Korean chemical bank, natural compound library, and personal chemical libraries; Supplementary Figure 1A and 1B). Among tested about 12000 chemicals, three chemicals (IPP-14, 22, and 23, IPP; Inhibitor of PAK1-PUMA) could block the interaction of PAK1 and PUMA (Supplementary Figure 1C). Thus, we checked the effect of these chemicals on cell viability of HCT116 and its isogenic HCT PUMA−/− cell lines [20]. Interestingly, IPP-14 (Supplementary Figure 1D), but not other chemicals (IPP-22 and 23), suppressed the cell viability in HCT116 (human colorectal cancer cells) (Figure 1A). However, HCT116 PUMA−/− showed the resistance to IPP-14-induced cell death (Figure 1A), indicating that IPP-14-induced cell death is achieved by PUMA-dependent manner. We also confirmed the inhibitory effect on PAK1-PUMA binding through Glutathione S-transferase (GST) pull-down and Immunoprecipitation (IP) assay (Figure 1B and Supplementary Figure 1E). To know how IPP-14 suppresses cell viability, we checked the expression of several related proteins by western blot analysis. Inconsistently with our expectation, IPP-14 did not alter the expression of Smad4 and PUMA, although PAK1 expression was reduced under serum-present condition (Figure 1C and 1D). Instead, we observed obvious induction of p21 in HEK293 and HCT116 cell lines (Figure 1C and 1D), differentially from commercial PAK1 kinase inhibitor, AG879 [21]. In addition, p53, well-confirmed p21 upstream regulator [22, 23], was not altered by IPP-14 (Figure 1D), suggesting that IPP-14 induced p21 is not related with p53.

### IPP-14 suppresses cancer cell viability and migration

To avoid the random cytotoxicity and false positive reaction of chemicals, we checked the cytotoxic effect of a group of chemicals including IPP-14 on PAK1 overexpressed pancreatic cancer cell line, MIA-Paca2 (Supplementary Figure 2A), comparing to human gastric cancer cell line, MKN45 (Supplementary Figure 2B). This analysis suggested that IPP-14 can obviously suppress cell viability in PAK1 overexpressed cells. IPP-14 showed more dramatic dosage effect on MIA-Paca2 viability than other chemicals (IPP-19, -23, and -27; Supplementary Figure 2C). Next, we checked the effect of IPP-14 on Smad4-deficient Capan-1 (human pancreatic cancer cells). As we expected, IPP-14 suppressed cell viability in the Smad4-deficient cells (Figure 1E). However, we did not observe the reduction of PAK1 expression or additional induction of PUMA expression by IPP-14 under the serum-free condition (Figure 1F). Instead, p21 induction was also detected in IPP-14-treated cells in regardless of Smad4 status (Figure 1F). Since PAK1 is involved in cell migration [3, 4], we next monitored the effect of IPP-14 on cell migration using transwell assay. Under low cytotoxic condition (1 μM, 16 hr), we could observe the inhibitory effect of IPP-14 on cell migration in HCT116 and Capan-1 (Figure 1G). Indeed, 1 μM of IPP-14 did not induce cytotoxicity at tested time points (From 12 hr to 18 hr; Supplementary Figure 3A and 3B).

### IPP-14 induces p21 expression

**Figure 2:**
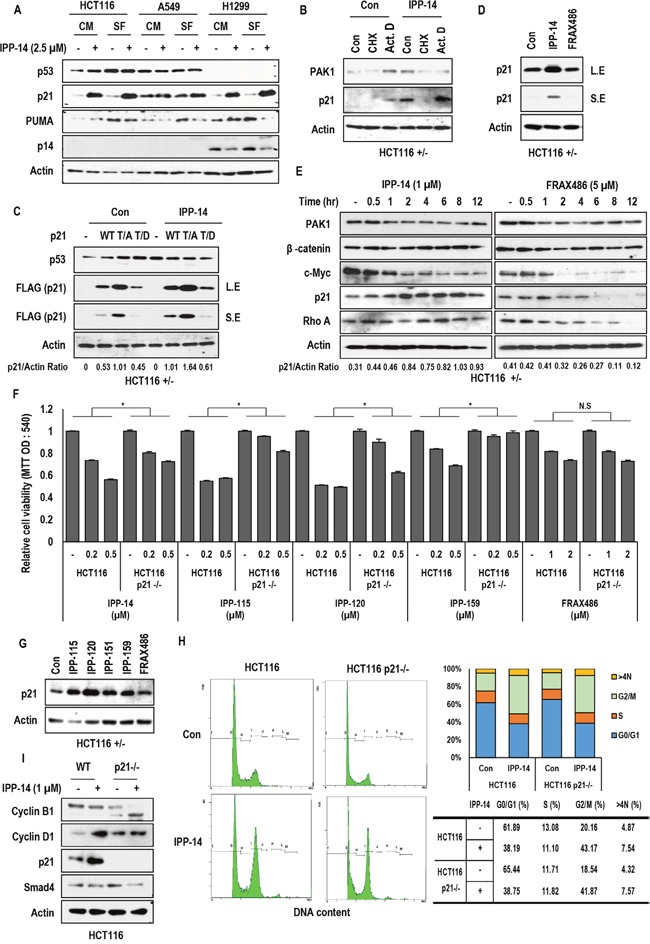
Rapid induction of p21 by IPP-14 **(A)** IPP-14 induces p21 expression in regardless of cell lines and serum condition. HCT116, A549 (human lung cancer cells), H1299 cell lines were treated with IPP-14 (2.5 μM) for 8 hr in serum-present or absent condition and western blot was performed using the indicated antibodies. Actin was used as loading control. **(B)** p21 induction is accomplished at post-translational level. Actinomycin D (Act. D; 1 μg/ml, Transcription inhibitor), Cyclohexamide (CHX; 100 μg/ml, Translational elongation inhibitor) were treated to block p21 induction by IPP-14. HCT116 cells were pre-treated Act. D or CHX for 2 hr before incubating with IPP-14 (1 μM). **(C)** Exogenous p21 is up-regulated by IPP-14. But p21-T145D mutant is induced marginally. HCT116 cells were transfected with p21 wild or mutant form (T145A, T145D), followed by treating IPP-14 (1 μM). Western blot was performed by using indicated antibodies. p21/Actin ratio was measured by using Image J software. **(D)** IPP-14 induces p21 expression but FRAX486 (known as selective PAK1 inhibitor) does not affect p21 upregulation. HCT116 cells were treated with IPP-14 or FRAX486 (5 μM). **(E)** p21 level is increased by IPP-14 but not by FRAX486. HCT116 cells were treated indicating chemicals for time-dependent manner and expression level was measured by western blot. p21/Actin ratio was measured by using Image J software. **(F)** Induction of cell death by IPP-14 and derivatives (IPP-115, 120, and 159) are not fully dependent on p21 induction. Moreover, FRAX486 does affect cell viability in regardless of p21 status. HCT116 and HCT116 p21-deficient cells were treated with indicating doses of chemicals for 48 hr, then cell viability was measured by MTT assay. *P< 0.005 (Student's *t*-test) **(G)** IPP-14 derivatives shows similar effect on p21 upregulation as IPP-14. HCT116 cells were incubated with IPP-14 derivatives (1 μM) or FRAX486 (5 μM) for 8 hr. **(H)** IPP-14 induces G2/M arrest in regardless of p21 status. Cells were treated with IPP-14 (1 μM) for 12 hr, followed fixing by PFA, and finally cell cycle was analyzed by FACS. **(I)** Inhibition of Cyclin B1 expression by IPP-14 in p21 deficient cells. Differentially from HCT116, where IPP-14 induced p21, HCT116 p21−/− cells showed the reduction of cyclin B1 in response to IPP-14 (1 μM). Western blot was performed by indicated antibodies. Actin was used as loading control.

Since induction of p21 is very obvious effect of IPP-14, we investigated the mechanism of it. First of all, we monitored the p21 induction in several kinds of cell lines. All tested cancer cell lines showed the induction of p21 in response to IPP-14 (Figure 2A and Supplementary Figure 4A) with very rapid kinetics (Supplementary Figure 4B) without transcriptional induction (Supplementary Figure 4C). In addition, *de novo* transcription inhibitor, Actinomycin D (Act. D) did not block the p21 induction (Figure 2B), indicating that IPP-14-induced p21 would be achieved by transcription independent mechanism. Moreover, *de novo* translation inhibitor, CHX, could completely eliminate p21 expression (Figure 2B), suggesting that IPP-14 might increase pre-existed p21 level. However, IPP-14 did not extend p21 half-life (Supplementary Figure 4D) and showed the additional effect with proteasome inhibitors (ALLN and MG132; Supplementary Figure 4E). These results indicated that there would be unusual regulation mechanism for p21 expression. To explore the mechanism of IPP-14-related p21 induction mechanism, we next checked the effect of IPP-14 on exogenous p21. IPP-14 could induce exogenous wild type p21 expression as well as T145D and T145A mutants (Figure 2C). However, the effect of IPP-14 on p21 mutants was less dramatic than that on wild type p21 (Figure 2C), implying that AKT-mediated p21 phosphorylation would be related with IPP-14 induced p21. Before testing the engagement of AKT on IPP-14 effect, we checked the involvement of PAK1 kinase activity. To test this, we measured the expression of p21 in the FRAX486 (selective PAK1 kinase inhibitor) [24, 25] treated cells. However, we did not observe the p21 induction (Figure 2D), despite long term treatment (Figure 2E). This result indicated that PAK1 kinase activity was not related with p21 induction by IPP-14. So, we returned to relevance of AKT on IPP-14 induced p21. Since p21-T145 residue is phosphorylated by AKT, resulted in rapid degradation of p21 [26], we monitored the effect of IPP-14 on AKT-PAK1 binding. Indeed, PAK1 N-terminal domain (not kinase domain) is associated with AKT [18]. Our GST pull down assay using PAK1-N-terminal domain showed the inhibitory effect of IPP-14 on the interaction of PAK1 and AKT1 (Supplementary Figure 4F). In addition, IPP-14 showed the similar effect on p21 expression with LY294002, PI3K inhibitor (Supplementary Figure 4G). Considering our result, IPP-14-induced p21 induction would be partially achieved by AKT1 suppression via PAK1-AKT binding inhibition. However, we did not fully demonstrate p21 induction by AKT1-PAK1 binding inhibition, because p21-T145D was also induced by IPP-14 (Figure 2C).

### The similar effect of IPP-14 derivatives on cell viability

To confirm the effect of IPP-14 and avoid the false positive effect, we obtained the IPP-14 derivatives from Korean chemical bank and checked the effect on cell viability (Supplementary Figure 5A). Among tested 100 relative chemicals, 4 chemicals (IPP-115, 120, 151, and 159) could suppress cell viability as strongly as IPP-14 (Supplementary Figure 5B) and showed the similar chemical structure (Supplementary Figure 5C). Since IPP-151 showed mild suppression effect on cell viability (Supplementary Figure 5B), we therefore tested 3 kinds of chemicals further study. In fact, three chemicals could induce p21 expression, like as IPP-14 (Supplementary Figure 5D). However, IPP-14 derivative did not alter the p21 half-life, similarly with IPP-14. (Supplementary Figure 5E; IPP-159). These results suggest that IPP-14 and its relative chemicals can show similar tumor suppressive function.

### The tumor suppressive effect of IPP-14 and its relatives is not fully dependent on p21

We next checked the cell viability in p21-deficient HCT116 after treatment of IPP-14 and its derivatives to know the dependency of p21. Indeed, HCT116 p21−/− [27] partially responded to IPP-14 related chemicals (Figure 2F). However, FRAX486 did not show the difference on cell viability between HCT116 and its isogenic HCT116 p21−/− (Figure 2F). This result indicated that IPP-14 and its derivatives could execute biological effect via different mechanism from PAK1 kinase activity inhibition. In fact, IPP-14 derivatives could induce p21 expression at post-translational level (Figure 2G and Supplementary Figure 5D). To address how IPP-14 could reduce cell viability in HCT116 p21−/− cells, we checked the cell cycle and found that IPP-14 could obviously induce G2/M arrest in regardless of p21 status (Figure 2H). Indeed, IPP-14 could suppress cyclin B1 expression in HCT116 p21−/− cells (Figure 2I). Our results indicated that IPP-14-mediated tumor suppressive effect was achieved by PUMA-mediated cell death (Figure 1A), p21-mediated cell cycle arrest (Figure 2F) and p21-independent G2/M arrest (Figure 2H).

### The biological effect of IPP-14 on PUMA deficient cells

**Figure 3:**
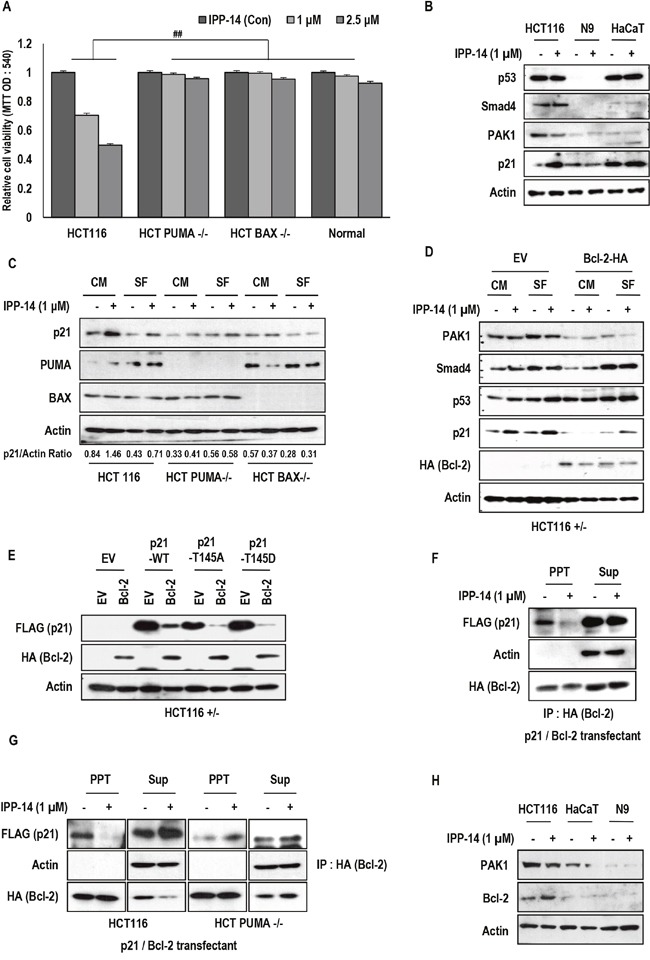
PUMA/BAX dependent effect of IPP-14 **(A)** IPP-14 does not show the effect of cell viability in isogenic PUMA/BAX deficient cell lines and normal fibroblast cells. After treatment of IPP for 48 hr, cell viability was measured by MTT assay. ## mean different group by ANOVA test (*P*< 0.001). **(B)** p21 induction by IPP-14 is not detected in normal fibroblast cells and HaCaT cells (Non-cancer cells). HCT116, Normal fibroblast, and HaCaT cells were treated IPP-14 (1 μM) for 8 hr and western blot was performed using the indicated antibodies. **(C)** IPP-14 increases p21 expression in HCT116 cells clearly, but not in PUMA/BAX deficient cells. HCT116 and PUMA or BAX deficient cell lines were treated with IPP-14 (1 μM) for 8 hr in serum-present (CM) or absent (SF) condition. p21/Actin ratio was measured by using Image J software. **(D)** Bcl-2 suppresses the IPP-14-induced p21. Moreover, Bcl-2 also suppresses p21 basal level. HCT116 cells were transfected with Bcl-2 (HA) for 24 hr, then IPP-14 (1 μM) was treated in serum contain or free condition. **(E)** Exogenous p21 expression is suppressed by Bcl-2 overexpression. HCT116 cells were co-transfected with Bcl-2 and p21 WT or MT (T145A and T145D) for 24 hr, then western blot was performed. **(F)** IPP-14 inhibits the interaction between Bcl-2 and p21. HEK293 cells, co-transfected with Bcl-2 and p21 for 24 hr, were incubated with IPP-14 (1 μM), and lysed for IP assay with anti-HA antibody. Actin was used as loading control and negative control. **(G)** PUMA is required for IPP-14-mediated inhibition of Bcl-2 and p21 binding. Under the same condition of above, IP assay was performed in HCT116, HCT PUMA deficient cells by anti-HA antibody. **(H)** Bcl-2 expression is low in normal fibroblast and HaCaT cells. HCT116, normal fibroblast, and HaCaT cells were exposed to IPP-14 (1 μM) for 8 hr.

Since PUMA-mediated cell death seemed to be one of important pathway of IPP-14-induced tumor suppression, we next checked the effect of IPP-14 in PUMA and BAX deficient cell lines [28]. Consistently with our previous result, IPP-14 only showed the marginal effect in PUMA deficient cells (Figure 3A). In addition, we did not observe obvious effect in HCT116 BAX−/− as well as normal human fibroblast (Figure 3A). In addition, we did not observe the induction of p21 in normal fibroblast and non-cancer HaCaT cells (human keratinocyte cells) (Figure 3B), suggesting that IPP-14 would be non-toxic on non-cancer cells. Next, we measured the expression of p21 in PUMA and BAX deficient cell lines. In the both cell lines, p21 induction was not obvious, comparing to parental HCT116 (Figure 3C). To confirm the independence of p53 on IPP-14-induced p21, we tested several cell lines. In regardless of p53 status, IPP-14 could induce p21 expression in all of tested cell lines (Supplementary Figure 6A), even in p53 deleted HCT116 (Supplementary Figure 6B). Since PAK1 kinase inhibitor, FRAX486 did not induce p21 expression (Supplementary Figure 6B), IPP-14-mediated p21 induction would be achieved via PAK1 kinase activity-independent mechanism. Indeed, elimination of PAK1 using siRNA did not induce p21 expression and eliminate IPP-14-mediated p21 induction (Supplementary Figure 6C). Thus, we focused on why PUMA/BAX deficient cells were resistant to IPP-14-induced p21 induction. IPP-14 and proteasome inhibitor did not show additional induction of p21 in PUMA and BAX deficient HCT116 cell lines comparing to HCT116 cells. (Supplementary Figure 4E and Supplementary Figure 6D). These results suggest that induction of p21 by IPP-14 was related with Bcl-2 family proteins. Since PUMA and BAX are pro-apoptotic protein and loss of them may release free Bcl-2 protein, we speculated that Bcl-2 would be inhibitor of p21. Actually, p21 expression was reduced in PUMA or BAX-deficient cells (Figure 3C). To confirm this, we measured the p21 expression in Bcl-2 transfected cells and found that Bcl-2 suppressed p21 expression and also blocked the IPP-14-induced p21 (Figure 3D). In addition, Bcl-2 reduced all kinds of exogenous p21 mutants (Figure 3E). Indeed, interaction between p21 and Bcl-2 was diminished by IPP-14 (Figure 3F). However, in PUMA deficient cells, the binding of p21 and Bcl-2 was not disrupted by IPP-14 (Figure 3G), indicating that IPP-14-mediated p21 induction would be achieved by inhibition of Bcl-2, which was facilitated by activated PUMA. In addition, expression of Bcl-2 was very low in normal fibroblast and non-cancer HaCaT cells, comparing to HCT116 (Figure 3H), provided the clue why these cells were resistant to IPP-14 induced p21. These results indicate releasing of p21 from Bcl-2 was one of important mechanism for inducing of p21 by IPP-14. However, until now, we did not have clear answer how Bcl-2 suppresses p21 expression, independently with proteasome.

### IPP-14 induces M-phase arrest by inhibition of PAK1

**Figure 4:**
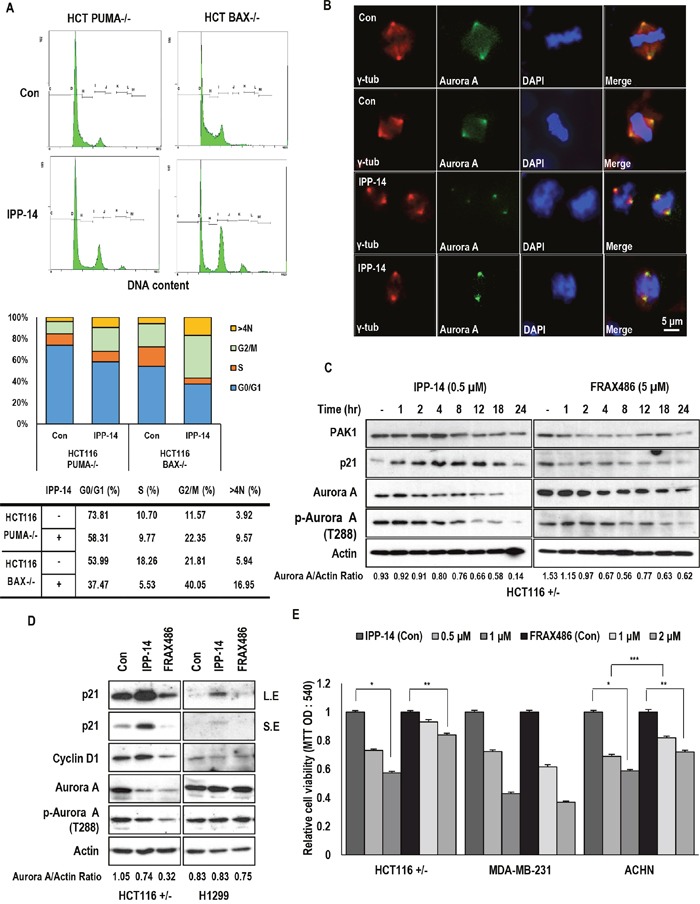
IPP-14 chemical induces G2 arrest through Aurora A inhibition **(A)** IPP-14 induces G2/M arrest in PUMA or BAX deficient cells. Cells were treated with IPP-14 (1 μM) for 12 hr, then cell cycle was analyzed by FACS. **(B)** IPP-14 inhibits the distribution of Aurora A following spindle fibers. HCT116 cells were incubated with IPP-14 (1 μM), immunofluorescence (IF) was performed by indicated specific antibody (Aurora A; Green, γ-tublin; Red) and DAPI (Blue). **(C)** Inhibition of Aurora A is detected earlier and at lower concentration by IPP-14 comparing to FRAX486. Chemicals were treated to cells in a time-dependent manner to monitor Aurora A suppression. Aurora A/Actin ratio was measured by using Image J software. **(D)** IPP-14 suppresses Aurora A and phosphorylation of Aurora A expression as FRAX486. HCT116 and H1299 cells were incubated with IPP-14 (1 μM) or FRAX486 (5 μM), then western blot was performed. Aurora A/Actin ratio was measured by using Image J software. **(E)** IPP-14 reduces cell viability in NF2 mutant cell lines. But effect of IPP-14 in MDM-MB-231 is not distinguishable comparing to FRAX486. Following treatment with the indicated concentration of IPP for 48 hr, cell viability was measured by MTT assay. *P< 0.005, **P = 0.008, ***P = 0.01. Statistical significance was calculated by Student's *t*-test.

Although we found that induction of p21 by IPP-14 was important for anti-tumor effect, p21 deficient cells still responded to IPP-14 (Figure 2F). These results indicated that there are additional mechanism for suppression of cancer cell viability. To reveal this, we checked the cell cycle in PUMA and BAX deficient HCT116 through FACS and observed the significant cell cycle arrest at G2/M phase (Figure 4A). Since the kinase activity of PAK1 facilitates M-phase progression through activation of Aurora A kinase [8], we assumed that IPP-14 also inhibits PAK1 kinase activity. Indeed, we found the blocking of BAD phosphorylation and the partial suppression of AKT phosphorylation by IPP-14 (0.5 μM), similarly with FRAX486 (5 μM), at low concentration (Supplementary Figure 6E). Furthermore, IPP-14 caused G2/M phase arrest (4N DNA) and increase of >4N DNA containing cell population (Figure 4A). Thus, we monitored the effect of IPP-14 on Aurora kinase A. The distribution of Aurora A following spindle fibers were abolished by IPP-14 (Figure 4B), which indicates that IPP-14 inhibits Aurora A kinase activity through PAK1. In addition, similarly with PAK1 inhibitor, FRAX486, IPP-14 obviously suppressed the Aurora A expression as well as phosphorylation of Aurora A in HCT116 (Figure 4C). However, in the H1299 cell (human lung cancer cells), reduction of Aurora A and induction of p21 were marginal (Figure 4D). Consistently, H1299 were resistant to IPP-14 (Supplementary Figure 6F). Until now, we did not know the reason of resistance but from this cell lines, we revealed that reduction of Aurora A and induction of p21 were important for biological effect of IPP-14. Comparing to FRAX486, IPP-14 could more rapidly suppress Aurora A at lower concentration (Figure 4C). Finally, we tested the effect of IPP-14 on NF2-deficient cancer cell lines, because NF2 suggested as PAK1 inhibitor [15, 16]. Compared to FRAX486, IPP-14 induced reduction of viability in two kinds of NF2-impaired cell lines, MDA-MB-231 (human breast cancer cells) and ACHN (human renal cancer cells) (Figure 4E). Since PAK1 inhibitors has been suggested as putative candidate for NF2 deficient cancers including NF2 syndrome, IPP-14 would be one of plausible chemicals for NF2 syndrome and other cancers. In addition, we tested the cooperative effect of IPP-14 with commercially used EGFR (Epidermal growth factor receptor) inhibitors. However, we only observed the IPP-14 effect (Supplementary Figure 6F).

## DISCUSSION

For a long time, inhibition of PAK1 has been suggested as one of plausible strategy for anti-cancer treatment [29, 30]. However, direct targeting of kinase activity has generated undesired side effect, because of similar structure of other kinase domain [29, 31]. In this study, we tried to find a specific inhibitor of PAK1-PUMA binding, because their binding occurs through N-terminal regulatory domain of PAK1. In addition, N-terminal region is important for association with AKT and NF2 [15]. Here, we suggested IPP-14 as specific inhibitor of PUMA and PAK1. Indeed, IPP-14 could suppress PAK1 kinase activity (Figure 1G) and induce M phase arrest via blocking of PAK1-mediated Aurora A activation (Figure 4A-4D). However, differentially from previous kinase inhibitor such as FRAX468, IPP-14 would be targeted to N-terminal regulatory domain of PAK1. Actually, in our previous result, PUMA is directly interacted with N-terminal PAK1 domain and we also used N-terminal region of PAK1 for drug screening. Although we did not provide the direct evidence for interaction of IPP-14 and PAK1 domain, considering that IPP-14 can block the interaction of PAK1 and AKT1 as well as PAK1 and PUMA. Its binding target would be N-terminal region of PAK1.

In addition, we observed the robust induction of p21 by IPP-14. Interesting it was independent with PAK1 and fully dependent on PUMA (Figure 3A-3C). Indeed, PUMA deficient cells were resistant to IPP-14-induced p21. Although we did not provide the underlying mechanism how Bcl-2 suppress p21, it is very clear that liberated p21 from Bcl-2 can induce cell cycle arrest and performed the tumor suppressive role and that IPP-14 can promote p21 releasing from Bcl-2. So, we are now investigating the molecular mechanism about Bcl-2-mediated p21 suppression.

**Figure 5:**
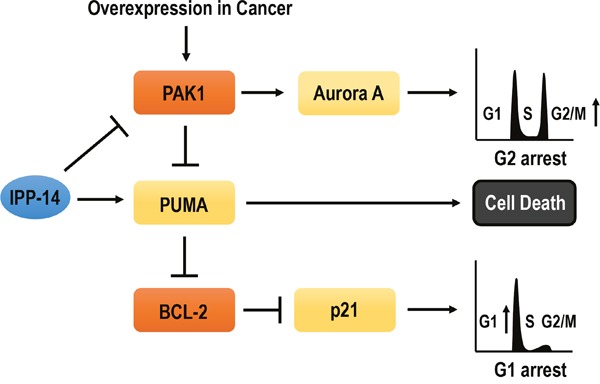
Diagram for summary IPP-14 inhibits PAK1 activity. It can induces G2/M phase arrest through Aurora A suppression and cell death through PUMA activation. Furthermore, IPP-14 can bind to PUMA as well as PAK1 then activated PUMA promotes p21 releasing from Bcl-2. However, p21 upregulation by IPP-14 is independent of PAK1 suppression. Thus, IPP-14 could be involved in PAK1-Aurora A mediated G2/M phase arrest, PUMA-mediated cell death, PAK1-independent p21 induction. So, It would be proper anti-cancer drug against PAK1 activated or Bcl-2 overexpressed cancers.

Although PUMA or BAX deficient cell lines are resistant to IPP-14-induced p21 induction, they are also arrested at G2/M phase by IPP-14 (Figure 4A), indicating that IPP-14-induced Aurora A inhibition was not related with PUMA. It would be achieved by suppression of PAK1 itself. Thus, IPP-14 could activate three signaling pathways, PUMA-mediated cell death, PAK1-independent p21 induction, and Aurora-mediated cell cycle arrest (Figure 5). This property seems to be very proper for using anti-cancer drug, since it can be used in apoptosis signaling defect cancer as well as cell cycle regulation defect cancers.

Moreover, IPP-14 worked very well at low concentration. This result indicates that IPP-14 is very specific inhibitor for PAK1 and Bcl-2. However, we did not fully demonstrate how IPP-14 can activate both signaling. Our hypothesis is that IPP-14 can bind to PAK1 as well as PUMA. Since PUMA is essential for p21 induction, we speculate that IPP-14 bound PUMA might associate to Bcl-2 and release p21 from Bcl-2-mediated p21 reduction. In other hands, IPP-14 associates with PAK1 N-terminal region and shut-down PAK1 activity. To address this, more intensive study should be performed.

Taken together, IPP-14, which has been isolated as PUMA-PAK1 binding inhibitor, can induce p21 and inhibit PAK1 activity. Thus, IPP-14 would be one of plausible anti-cancer drug candidate against PAK1 activated or Bcl-2 overexpressed cancers.

## MATERIALS AND METHODS

### Cell culture and reagents

HCT116 (p53+/−) cells and its isogenic cell lines (p53−/−, p21−/−, PUMA−/− and BAX−/−) were provided by Dr. B. Vogelstein (Johns Hopkins University) [20, 27, 28, 32]. Human cell lines used in this study were obtained from the American Type Culture Collection (ATCC, Manassas, VA, USA) and the Korean Cell Line Bank (KCLB, Seoul, South Korea). Cell lines were maintained in liquid media containing 10% fetal bovine serum and 1% penicillin-streptomycin (RPMI-1640 or DMEM), at 37°C and 5% CO2. Human fibroblast cell (9-year-old female) was obtained from the Coriell Cell Repositories (New Jersey, USA) and maintained in EMEM, containing 15% FBS, 2 mM Glutamine with 26 mM HEPES without antibiotics. For serum starvation, cell lines were incubated with serum deprivation (SF; Serum Free) media for indicating times. Cyclohexamide (CHX; C4859) was purchased from Sigma. AG879 (658460), Actinomycin D (114666) was obtained from Calbiochem (Darmstadt, Germany), and Okadaic acid (ALX-350-003) was purchased from Enzo Life Science (Farmingdale, NY, USA). FRAX486 was purchased from Chemitek (Indianapolis, IN, USA).

### Chemical screening

For chemical screening, we generated ELISA assay system [19]. To isolate PAK1-PUMA binding inhibitor, we immobilized PAK1 recombinant protein on a 96-well plate using 0.5% paraformaldehyde (PFA). After the plates were dried and washed with phosphate-buffered saline (PBS), we incubated with 25 μM, 50 μM of chemicals (final concentration), followed by adding PUMA protein. After 2 hr reaction, the 96-well plates were washed with PBS and blocked by 3% skim milk to remove background. The plates were incubated with anti-PUMA Ab (diluted in 1:10,000) for 1 hr and then anti-rabbit IgG-HRP (diluted in 1:50,000) for 1 hr. After washing twice, plates were incubated with a 3,3′,5,5′-tetramethylbenzidine (TMB) solution (Calbiochem) for 30 min and Stop solution (1N H_2_SO_4_) for 30 min. Finally, we speculated the value by using the ELISA reader (absorbance at 450 nm). Chemical libraries were provided by GY Song (Chungnam National University, Deajeon, Korea), HY Moon (Pusan National University, Busan, Korea) and Korean Chemical Bank (Korea). About 12,000 chemical compounds were used in this screening.

### Recombinant proteins

To produce the recombinant proteins, the human PAK1 N-terminal domain fragment (residue 70-149) and the full-length human PUMA (residue 1-193) were ligated into the *Eco* RI and HindIII sites of the pGEX-TEV vector, which is modified vector made by adding a TEV protease cleavage site to pGEX-4T1 (Invitrogen). The recombinant proteins were expressed in the Escherichia coli (E.*coli*) strain BL21 (DE3) as GST-fusion proteins. The proteins were purified by glutathione-affinity chromatography.

### Immunoblotting and protein-protein interaction analyses

Proteins were extracted from cells with RIPA buffer (50 mM Tris-Cl, pH 7.5, 150 mM NaCl, 1% NP-40, 0.1% SDS and 10% sodium deoxycholate). Samples were separated via SDS-PAGE and transferred to PVDF membrane. Blotted membranes were blocked by 3% skim milk for 1 hr and incubated with specific antibodies. The following antibodies were used in this study; p21 (sc-397), Smad4 (sc-7966), Actin (sc-1616), HA (Hemagglutinin; sc-7392), GST (sc-138), GFP (Green fluorescent protein; sc-8036), and p53 (DO-1) (sc-126) were purchased from Santa Cruz biotechnology (Santa Cruz, CA, USA). PUMA (4976), PAK1 (2602), Aurora A (14475), p-Aurora A (Thr288) (3079) were obtained from Cell Signaling Technology (Danvers, MA, USA). Anti-FLAG (Sigma; F3165) was provided by Sigma Aldrich (St, Louis, Mo, USA), HRP-conjugated goat anti-mouse, goat anti-rabbit and mouse anti-goat antibodies (Pierce, Thermo Fisher Scientific, Inc., Rockford, IL, USA) were used as secondary antibodies. For the analysis of protein-protein interaction, Glutathione S-transferase (GST) pull-down assay and Immunoprecipitation (IP) experiment were performed. To detect the interaction between PUMA and PAK1 or AKT and PAK1, GST pull-down assay was conducted using agarose bead-conjugated GST-PAK1-N terminal domain (residues 70-149). After incubating GST-recombinant protein with transfected lysates (PUMA or AKT) for 1 hr at 4°C, the precipitated proteins were also separated by SDS-PAGE and analyzed via western blot experiment. IP assay were performed by using a proper antibody and with transfected lysates. Whole cell lysates expressing p21-FLAG and Bcl-2-HA were incubated with anti-HA antibody for 2 hr at 4°C and then with protein A/G agarose beads (Invitrogen, Carlsbad, CA, USA) for 2 hr. After centrifugation and washing with RIPA buffer, the immunocomplexes were separated by SDS-PAGE and subjected to western blot with anti-FLAG, Actin and HA.

### Immunofluorescence staining

Cells on coverslips were washed with PBS and fixed with 4% PFA for 30 min at room temperature and then permeabilized in 0.1% Triton X-100/PBS for 10 min. After cells were treated with blocking solution (anti-Human Antibody diluted 1:500 in PBS) for 1 hr, cells were incubated with anti-Aurora A (diluted in 1:200), γ-tublin (T6557; Sigma; diluted 1:500 in blocking solution) for overnight at 4°C. Finally, the cells were incubated with FITC and Rhodamine-conjugated secondary antibodies at 4°C for 6 hr. The nucleus was stained with 4, 6-diamidino-2-phenylindole (DAPI) for 10 min. Cells were washed three times with PBS, then the coverslips were mounted with mounting solution (H-5501; Vector Laboratories (Burlingame, CA, USA)) and analyzed by fluorescence microscopy (Zeiss Axioplan2, Oberkochen, Germany), 400x magnification.

### FACS analysis

To analyze cell cycle, cells were seeded on 6 well plates and incubated with or without indicated chemical 12 hr. After washing with PBS, cells were fixed with 70% ethanol for 2 hr at 4°C. After fixation, cells were re-suspended in PBS containing 50 μg/ml propidium-iodide (P4170; Simga Aldrich) and 10 μg/ml RNaseA (Novagen) for 20 min. Approximately 10,000 cells were sorted by FACS (FC500; Beckman coulter) with an argon laser (488 nm) and analyzed by CXP software 2.0

### Transfection of vectors and si-RNA

HA-Bcl-2 expression vector was kindly provided by Dr. Ha, NC (Seoul National University). FLAG-p21, T145A, T145D expression vectors were purchased from Addgene (Cambridge, MA, USA). For *in vitro* gene knock down, si-RNAs against PAK1 and a non-target si-RNA (si-con) were obtained from Cosmogene Tech. Target sequence of si-PAK1 is followed; PAK1 no. 1: 5′-ACCCAAACATTGTGAATTA-3′; PAK1 no. 2: 5′- GGAGAAATTACGAAGCATA-3′; PAK1 no. 3: 5′-TCTGTATACACACGGTCTG-3′. Transfection was performed using Jet-PEI reagent (JetPEI; Polyplus transfection, New York, NY, USA) according to the manufacturer's protocol.

### RNA isolation and RT-PCR

For RT-PCR, total cellular RNA was extracted using RNA extraction kit (Qiagen). After measurement of RNA concentration, 1 μg of total RNA was reverse transcribed to cDNA using MMLV RT (Invitrogen) and random hexamers. RT-PCR was performed with the following specific primers: p21, (5′-CGTGAGCGATGGAACTTCGAC-3′ and 5′-GAT GTAGAGCGGGCCTTTGAG-3′) or PUMA, 5′-GGGG ACTTTCTCTGCACCATG-3′ and 5′-CACCAGCACA ACAGCCTTTCC-3′) and GAPDH (5′-ATCTTCCAGGA GCGAGATCCC-3′ and 5′- AGTGAGCTTCCC GTTCA GCTC-3′).

### Measurement of cell viability

To examine the cell viability, cells were incubated with 0.5 mg/ml of MTT solution (475989; Merck, Darmstadt, Germany) for 4 hr at 37°C. After removing excess solution and washing with PBS, the precipitated materials were dissolved in 200 μl DMSO and quantified by measuring absorbance at 540 nm.

### *In vitro* migration assay

For the analysis of *in vitro* cell migration, Transwell assay was performed using a pore size 8 μm Polycarbonate Membrane Transwell Inserts (3422; Corning, NY, USA). First, 0.6 ml media containing 10% FBS was added to the well plate. Cells were re-suspended in serum-free medium, and 0.1 ml of the cell suspension was added to the inside compartment. The plate was incubated with or without indicated chemical in 5% CO_2_ incubator for 16 hr. Then the cells in upper chamber were removed, and the attached cells in the bottom section were fixed by 4% PFA for 30 min. After fixation, migratory cells are stained by 0.5% Trypan blue solution (Gibco, BRL, Paisley, UK) and quantified. The migration rate was quantified by counting the migration cells in six random fields using a light microscope.

### Statistical analysis

The student's *t*-test was used for comparisons of two groups. *P*-value less than 0.05 was considered significant. Error bars indicate standard deviation. For ANOVA (Analysis of Variance) test, numeric variables were summarized by their mean±SD (standard deviation). Before ANOVA for group comparison is performed, we first applied Shapiro-Wilk test and Levene's test to check data normality and to assess the equality of variances, respectively. When both normality and homoscedasticity are met, one-way analysis of variance was conducted to compare the difference of a response variable for each condition, and a Tukey's HSD test was performed for post-hoc multiple comparison. When the data are satisfied with neither normality nor homoscedasticity, a non-parametric method Kruskal-Wallis test was used to assess equality of multiple group means, and then Mann-Whitney U test was performed for post-hoc multiple comparisons. For adjusting family-wise Type I error rate, we applied Bonferroni correction to adjust the p-values of multiple testing. SPSS 21.0 was used for all statistical analysis.

## SUPPLEMENTARY MATERIALS FIGURES AND TABLES


