# Supplementary material for: Anti-cancer effect of novel PAK1 inhibitor via induction of PUMA-mediated cell death and p21-mediated cell cycle arrest
Source: Oncotarget. 2017 Feb 28;8(14):23690–701. doi: 10.18632/oncotarget.15783 (PMC5410337; doi:10.18632/oncotarget.15783)
Supplement: Supplementary file 1 [file oncotarget-08-23690-s001.pdf]

Anti-cancer effect of novel PAK1 inhibitor via induction of PUMA-mediated cell death and p21-mediated cell cycle arrest

SUPPLEMENTARY FIGURES

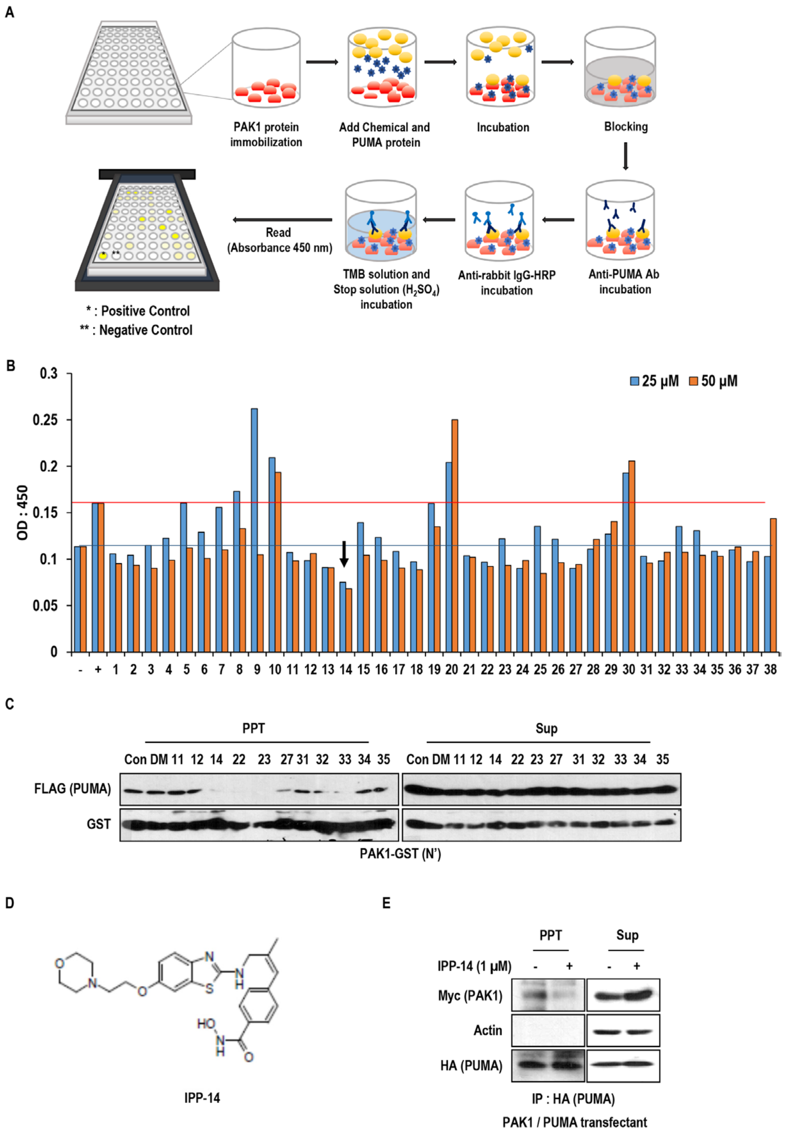

Supplementary Figure 1: Screening PAK1-PUMA binding inhibitor. (A) Schematic diagram of ELISA based chemical screening. PAK1 recombinant protein was fixed in a 96-well plate and incubated with PUMA protein and chemicals. After 2 hr incubation, plates were washed with TBST and treated with blocking solution. After washing, plates were incubated with anti-PUMA antibody and anti-rabbit-IgG-HRP. After washing twice, plates were incubated with TMB solution and Stop solution. Finally, plates were speculated by using ELISA reader (absorbance at 450 nm). For the detail information about the ELISA system were provided in material and method. **(B)** ELISA based screening experiments are performed by using 3 kinds of chemical libraries to random screening. Negative control (-; blue line) was incubated without PUMA protein and chemical and positive control (+; red line) was incubated with PUMA protein without chemical. **(C)** Interaction of PAK1 and PUMA is inhibited by IPP-14, 22, 23. GST-PAK1 (N-terminal) recombinant proteins and PUMA-FLAG overexpressing HEK293 cell lysates were incubated with or without selected chemical candidates. Then, GST Pull-down assays were performed. PPT indicated precipitated proteins, and Sup indicates supernatant. **(D)** Chemical structure of IPP-14. **(E)** IPP-14 disrupts the binding between PAK1 and PUMA. For IP analysis, HEK293 cells were transfected with PAK1 and PUMA vector for 24 hr and incubated with IPP-14 for 8 hr. Then, IP analysis was performed with anti-HA.

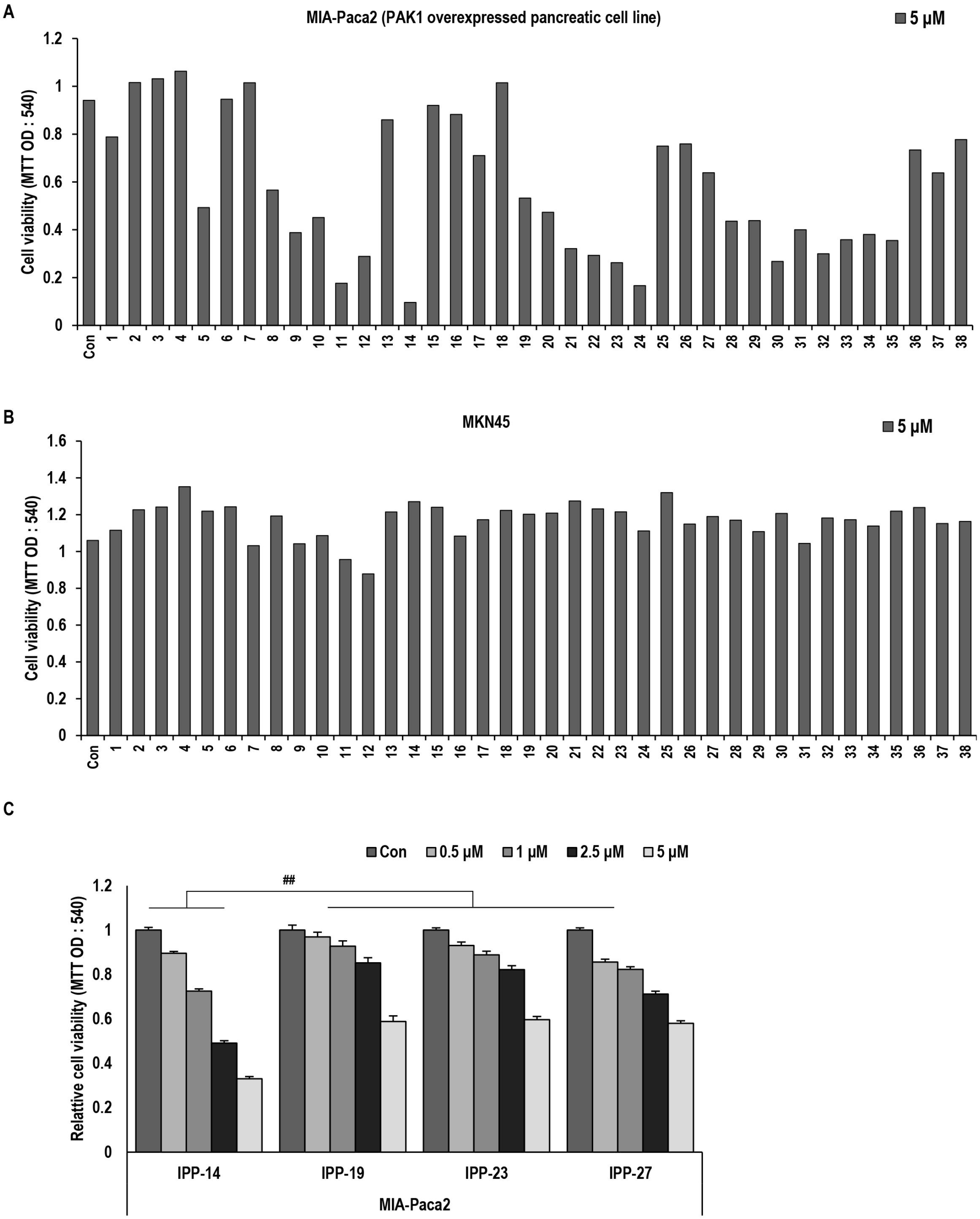

Supplementary Figure 2: Effect of PAK1-PUMA binding inhibitor. **(A–B)** PAK1 overexpressed cell lines show sensitivity to IPP-14. IPP-14 suppressed the viability of MIA-Paca2 (PAK1 overexpression; A), but not in MKN45 (Negative control; B). Following treatment with IPP (5 μM) for 48 hr, cell viability was measured by MTT assay. **(C)** IPP-14 induces cell death effectively at low concentration, compared to other chemicals, in MIA-Paca2. The MTT assay was performed to measure cell viability following 48 hr of treatment of chemicals. ## mean different group by ANOVA test ($P < 0.001$).

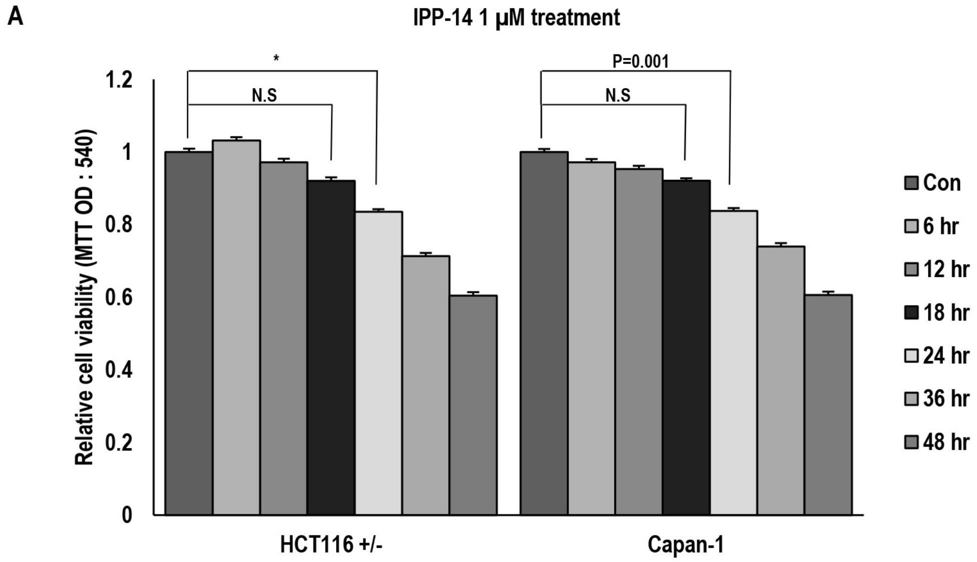

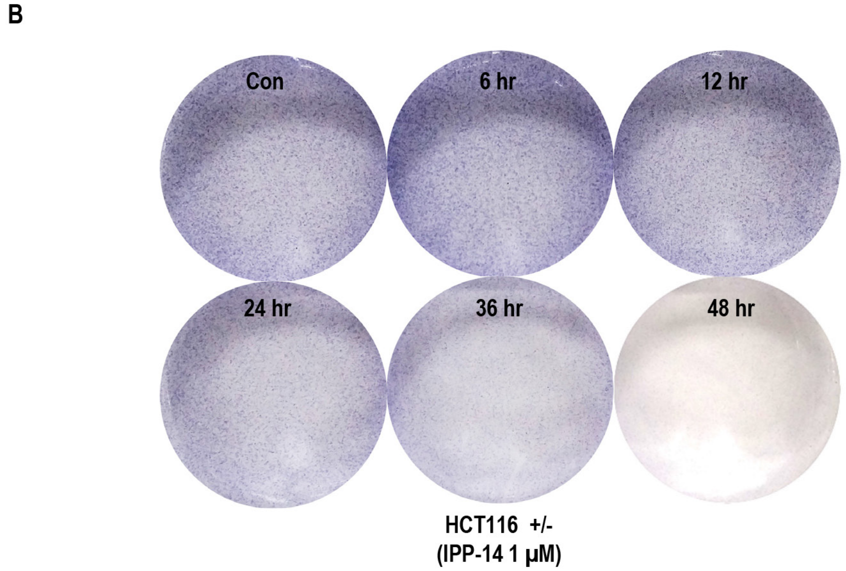

Supplementary Figure 3: The effect of IPP-14 on cell viability. (A) IPP-14 suppresses the viability of HCT116 and Capan-1 cells in late times. IPP-14 showed marginal effect on a cell viability at early times. But cell viability was remarkably decreased at 48 hr. HCT116 and Capan-1 cells were treated with IPP-14 for time-dependent manner. The MTT assay was performed to measure cell viability following treatment of chemicals. **(B)** Sensitivity of IPP-14 on cell viability effects. HCT116 cells were treated with 1 μM IPP-14 for 2 days. After PFA fixation, cells were stained with trypan blue.

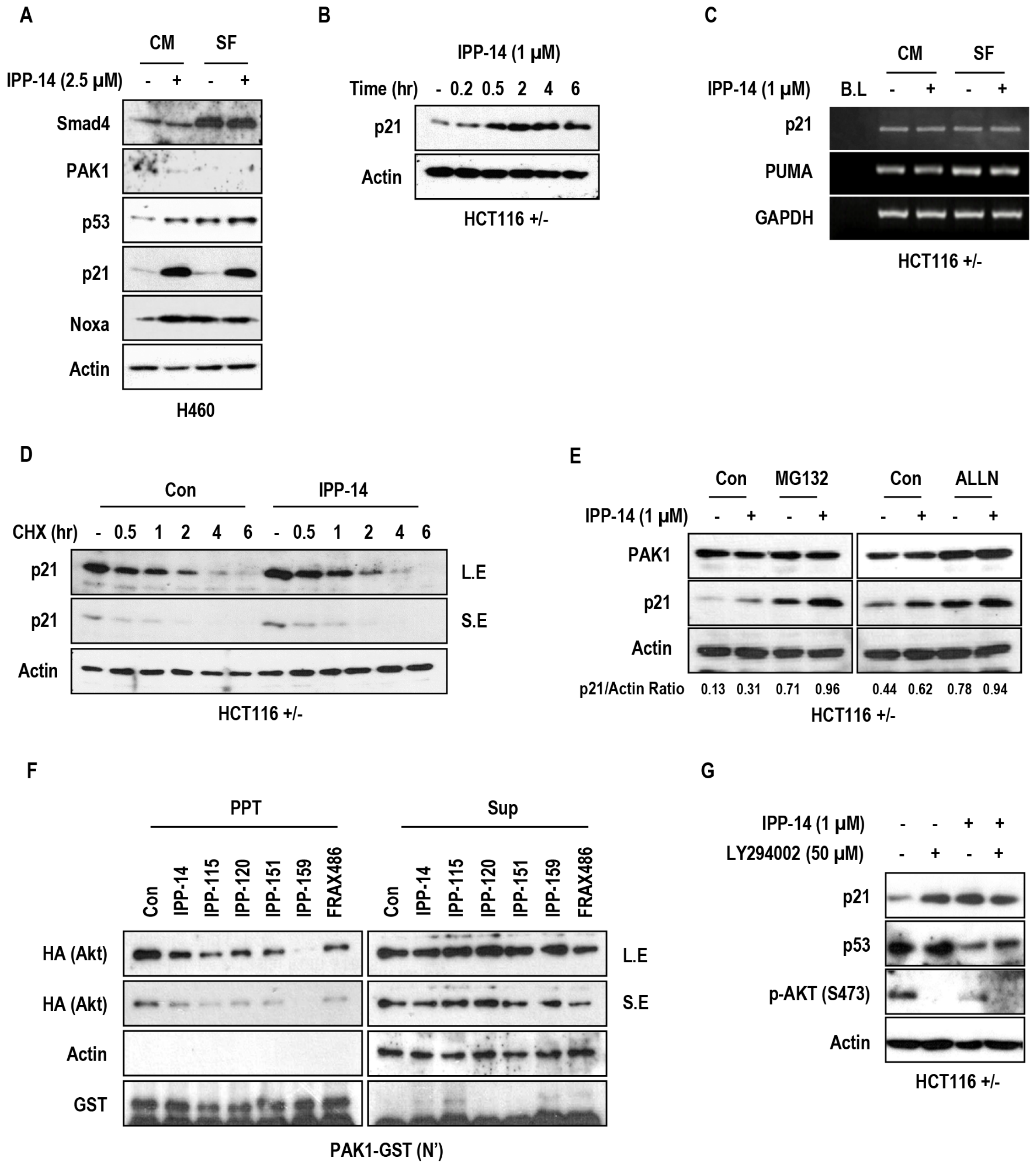

Supplementary Figure 4: Induction of p21 by IPP-14. **(A)** p21 is induced by IPP-14 in regardless of serum presence. H460 (human lung cancer cells) were treated with IPP-14 (2.5 µM) in serum containing (CM) or deprivation conditions (SF) for 8 hr. Western blot was conducted by using indicated antibodies. Actin used as loading control. **(B)** IPP-14 rapidly induces p21 expression. HCT116 cells were incubated with IPP-14 (1 µM) for indicated time and subjected to WB analysis. **(C)** IPP-14 do not induce p21 expression at mRNA level. RT-PCR analysis of the indicated genes was performed in HCT116 cells after treatment with IPP-14 (1 µM) for 8 hr. B.L indicates blank. **(D)** IPP-14 increases p21 expression but not stability. HCT116 cells were treated with Cyclohexamide (CHX; 100 µg/ml, Translational elongation inhibitor) for indicated times with or without IPP-14 (1 µM). **(E)** IPP-14 and proteasome inhibitor show additional effect in p21 induction. HCT116 cells were incubated with IPP-14 (1 µM) and MG132 (10 µM) or ALLN (10 µM) for 8 hr. p21/Actin ratio was measured by using Image J software. **(F)** Interaction of PAK1 and AKT is inhibited by IPP-14 and derivatives (2.5 µM). GST-PAK1 (N-terminal) recombinant protein was incubated with lysates from HEK293 cells transfected with AKT-HA and indicated chemicals. L.E indicates long exposure and S.E indicates short exposure. **(G)** Induction of p21 by IPP-14 is similar to PI3K inhibitor. LY294002 (50 µM) was treated with or without IPP-14 (1 µM) for 8 hr.

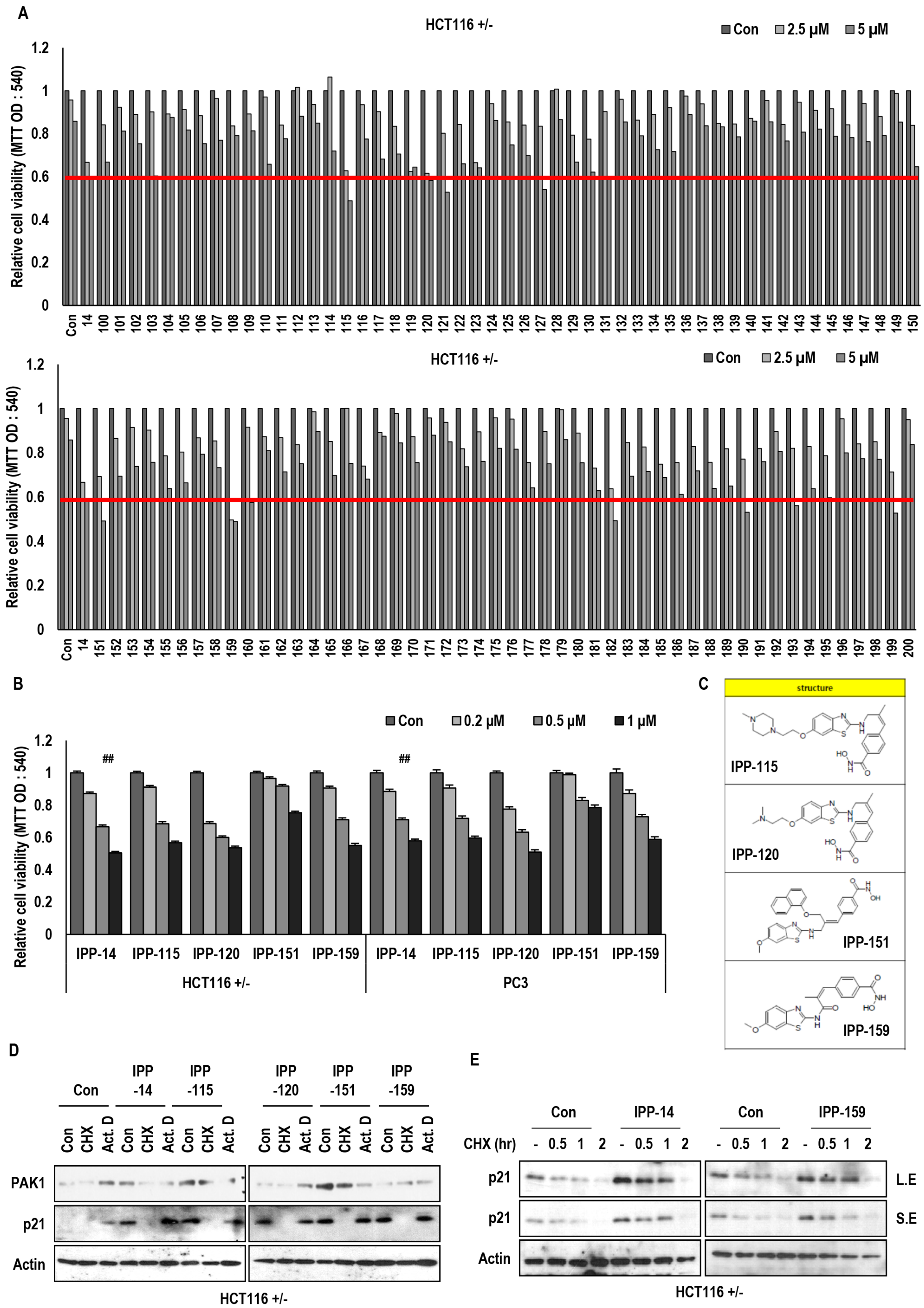

Supplementary Figure 5: Effect of IPP-14 derivatives. **(A)** IPP-14 and several kinds of derivatives decrease HCT116 cells viability. Among tested 200 IPP-14 derivatives, several chemicals suppressed the cell viability. **(B)** IPP-115, 120, and 159 suppress HCT116 and PC3 (human prostate cancer cells) viability as strongly as IPP-14. Based on Supplementary Figure 5A, four chemicals were tested their activity on two human cancer cell lines through MTT assay after incubation with indicated concentration of chemicals for 48 hr. ## mean different group by ANOVA test ($P < 0.001$). **(C)** Chemical structures of IPP-14 derivatives (IPP-115, 120, 151, and 159). **(D)** IPP-14 derivatives work as IPP-14 at post-translational level about p21 induction. Actinomycin D (Act. D; 1 μg/ml), Cyclohexamide (CHX; 100 μg/ml) were treated to block p21 induction by IPP-14 or derivatives. HCT116 cells were pre-treated Act. D, CHX for 2 hr before incubating with IPP-14 or derivatives (1 μM). **(E)** IPP-14 derivative does not extend p21 stability like as IPP-14. HCT116 cells were treated with CHX for indicated times with or without IPP-14 and IPP-14 derivative (IPP-159; 1 μM).

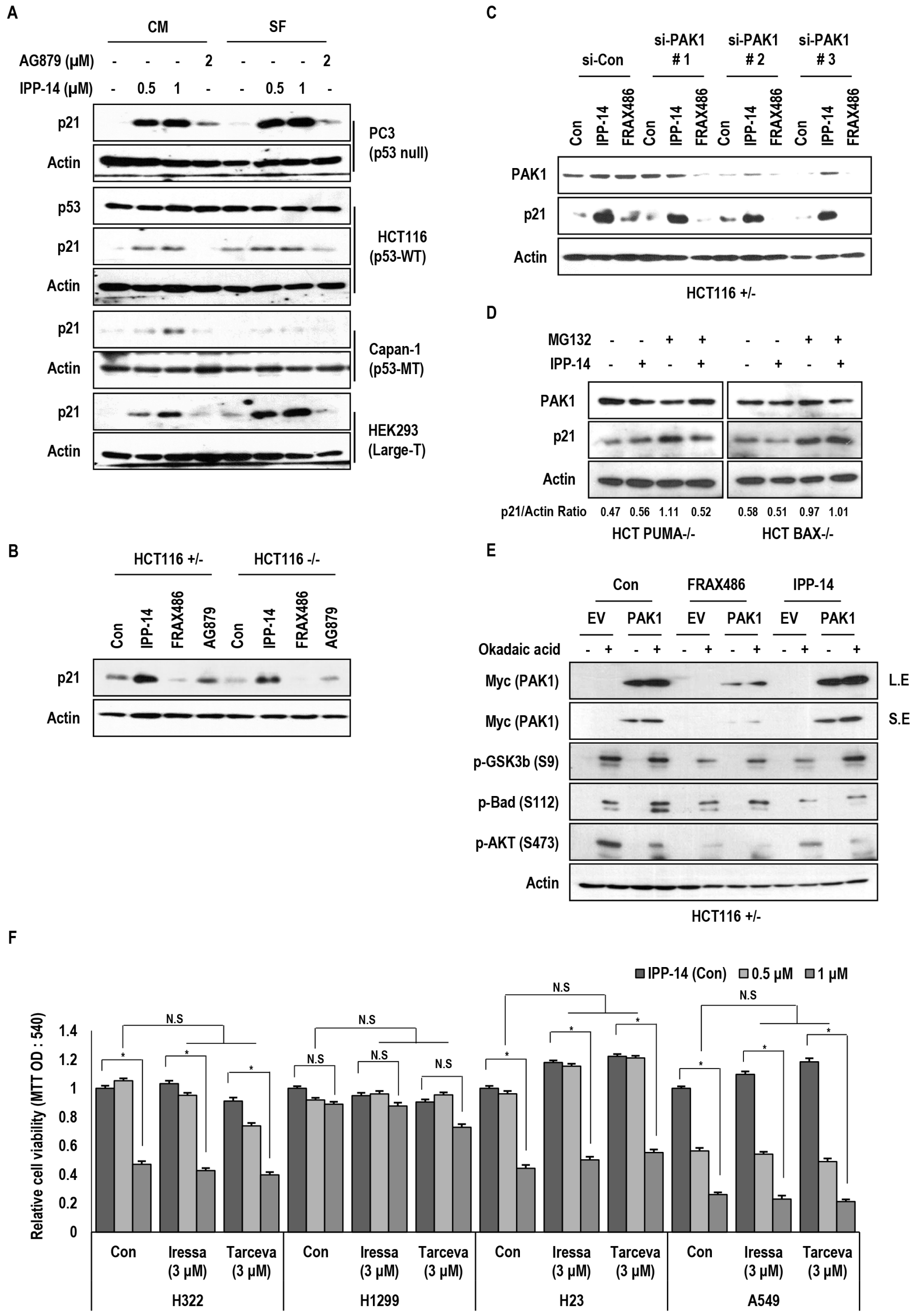

Supplementary Figure 6: p21 induction by IPP-14 is not related PAK1 or p53. **(A)** Upregulation of p21 by IPP-14 is independent of p53 status. (PC3; p53 null, HCT116; p53 WT, Capan-1; p53 MT, HEK293; p53 inactivation). Cells were treated with indicating concentration of IPP-14 or AG879 in serum containing (CM) or deprivation conditions (SF) for 8 hr. Western blot was performed by using the indicated antibodies. **(B)** PAK1 inhibition do not induce p21 expression. HCT116 cells were treated with indicated chemicals for 8 hr. (IPP-14; 1 μM, FRAX486; 5 μM, and AG879; 10 μM). **(C)** IPP-14 increases p21 expression in regardless of PAK1. HCT116 cells were transfected with si-PAK1 for 24 hr, followed by incubating with IPP-14 (1 μM) or FRAX486 (5 μM). **(D)** IPP-14 and proteasome inhibitor do not show additional induction of p21 in PUMA or BAX deficient cells. HCT PUMA or BAX deficient cell lines were treated with IPP-14 (1 μM) and MG132 (10 μM) for 8 hr. p21/Actin ratio was measured by using Image J software. **(E)** IPP-14 inhibits PAK1 kinase activity. HCT116 cells were treated with IPP-14 (1 μM) or FRAX486 (5 μM). Okadaic acid (OA; 100 nM, protein phosphatase inhibitor) was co-treated with IPP-14 and FRAX486 for 6 hr. **(F)** IPP-14 induces cell death in several lung cancer cell lines excepting H1299 independent manner of EGFR inhibitor. H322, H23, A549, H1299 (human lung cancer cells) were treated with IPP-14 and EGFR inhibitor (Iressa, Tarceva). Following treatment with the indicated concentration of chemicals for 48 hr, cell viability was measured by MTT assay. *$P < 0.005$ (t-test).
